# Using mobile technology for effective detection and prevention

**DOI:** 10.1186/2047-2994-4-S1-P252

**Published:** 2015-06-16

**Authors:** S Batra, S Ahuja, V Lauria, N Harshe

**Affiliations:** 1 Operation ASHA, India

## Introduction

There are 1.8 million new cases of TB in India each year. Open cases of TB infect 10-15 others annually. Close contacts of patients are likely to contract TB. It is also well known that there are 700,000 undetected patients in India and three million across the world.

## Objectives

An urgent need is to scale up detection of these hidden patients. The eDetection App developed by Operation ASHA enables providers to systematically screen at-risk individuals, thus effectively curbing an exponential spread of TB using an extremely cost-effective model.

## Methods

The eDetection App runs on tablets. It was piloted on 1 tablet in 1 city in India, Gwalior. During the 12 month pilot, the community health worker visited families of existing patients, factories where patients work and also went door-to-door in slum communities that have a high prevalence. He used the App to engage the community members and ask them answers to a basic questionnaire, and subsequently facilitate testing and diagnosis of patients.

## Results

number, 134 tested positive for TB and have been subsequently enrolled in TB treatment program. The total cost for the intervention was $2388, (community health worker salary $159x12; training of the CHWs $159; cost of hardware for eDetection $121, license fee for one copy of the software $200 - This is the same license fee that is charged from third parties using the software). The cost of detecting a patient of TB was $18. Comparable cost in a program in South Africa was $1,117 [1]. This is higher than the per patient cost with eDetection by 62 times. This proves that eDetection is not only effective in improving detection, but it has huge potential in saving costs. Implementation of eDetection system across the world could lead to a reduction for the cost of detection of the 3 million undognised cases by $3.3 billion, to only $54 million. Financials savings are apart from incredible improvement in patient care and community life, caused by early detection.

## Conclusion

The eDetection App is a simple and effective tool for finding hidden TB cases in the community and it is extremely cost-effective. Further large scale interventions are now needed to scale and replicate this technology to identify the hard-to-reach TB patients.

## Disclosure of interest

None declared.
